# Novel Therapeutic Strategies for Ischemic Stroke: Recent Insights into Autophagy

**DOI:** 10.1155/2022/3450207

**Published:** 2022-06-08

**Authors:** Xiaocheng Lu, Jian Zhang, Yu Ding, Jiang Wu, Gang Chen

**Affiliations:** ^1^Department of Neurosurgery, Brain and Nerve Research Laboratory, The First Affiliated Hospital of Soochow University, Suzhou, China; ^2^Department of Neurosurgery, The Affiliated Suqian Hospital of Xuzhou Medical University, No.138, Huanghe South Road, Suqian, 223800 Jiangsu, China

## Abstract

Stroke is one of the leading causes of death and disability worldwide. Autophagy is a conserved cellular catabolic pathway that maintains cellular homeostasis by removal of damaged proteins and organelles, which is critical for the maintenance of energy and function homeostasis of cells. Accumulating evidence demonstrates that autophagy plays important roles in pathophysiological mechanisms under ischemic stroke. Previous investigations show that autophagy serves as a “double-edged sword” in ischemic stroke as it can either promote the survival of neuronal cells or induce cell death in special conditions. Following ischemic stroke, autophagy is activated or inhibited in several cell types in brain, including neurons, astrocytes, and microglia, as well as microvascular endothelial cells, which involves in inflammatory activation, modulation of microglial phenotypes, and blood-brain barrier permeability. However, the exact mechanisms of underlying the role of autophagy in ischemic stroke are not fully understood. This review focuses on the recent advances regarding potential molecular mechanisms of autophagy in different cell types. The focus is also on discussing the “double-edged sword” effect of autophagy in ischemic stroke and its possible underlying mechanisms. In addition, potential therapeutic strategies for ischemic stroke targeting autophagy are also reviewed.

## 1. Introduction

Stroke, also known as cerebrovascular accident, is one of the leading causes of death worldwide, and survivors frequently experience long-term cognitive and functional limitations. Stroke can be classified as two main types: hemorrhagic stroke and ischemic stroke, of which, the later one is the common type of stroke, due to insufficient blood supply to the brain. To date, intravenous recombinant tissue plasminogen activator (rtPA) is the only Food and Drug Administration (FDA)-approved treatment strategy for acute ischemic stroke [1, 2]. Intravenous thrombolysis has been proven to be a relatively safe and efficient treatment; however, there are still some limitations, such as the therapeutic window which is 3-4.5 h following symptom onset and it may cause intracerebral hemorrhage [2, 3]. Thus, cutting-edge investigations on novel treatment strategies for ischemic stroke are urgently needed.

Autophagy, first defined by De Duve, is an evolutionarily conserved process for degrading and recycling of unnecessary or dysfunctional cellular components and proteins [4]. It is activated in response to stress conditions, such as nutrient limitation and oxidative stress. Autophagy has been shown to play a variety of pathophysiological roles in many disease, including neurodegenerative disease, liver disease, aging, and cardiovascular disease [5–8]. Moreover, autophagy is extensively observed in ischemic brain tissues, and the roles of autophagy in the ischemic stroke have been widely studied [9]. However, to date, the exact roles of autophagy in ischemic stroke are still controversial. Therefore, this review aims to focus on the function of autophagy in ischemic stroke, including in modulation of microglial phenotypes, activation of astrocytes, neuronal cells death, and blood-brain barrier (BBB) permeability. We will also discuss the possibility of treatments targeting autophagy as novel therapeutic strategies for ischemic stroke in the future.

## 2. Autophagy: An Overview

Autophagy is a cellular degradation machinery which is evolutionarily conserved from yeast to mammals and plays an important role in differentiation, development, and cellular homeostasis [10, 11]. It is activated by nutrient limitation or metabolic stress to degrade misfolded proteins or damaged organelles into metabolic elements. Dysregulation of autophagy is observed in a wide range of pathological conditions, including Alzheimer's disease, breast cancer, kidney disease, and inflammatory bowel diseases. [12–16] The known classes of autophagy in mammalian cells are macroautophagy, microautophagy, and chaperone-mediated autophagy.

### 2.1. Macroautophagy

Macroautophagy, initially studied in yeast and conserved across evolution, is characterized by sequestration of cytoplasmic material, such as organelles, protein aggregates, and lipid droplets, in a double-membrane-bound vacuole called the autophagosomes in a selective way. Subsequently, the autophagosomes deliver cargo to the lysosome for final degradation [17, 18]. For selective macroautophagy, autophagy adaptors (such as P62, NBR1, NDP52, and OPTN) specifically target cellular cargo to the autophagosomes for autophagy in an ubiquitin-dependent (Ub-dependent) or Ub-independent pathway [19–21]. Several protein complexes have been reported to be essential for the formation of autophagosome, including unc-51 like autophagy activating kinase 1 (ULK1) complex, microtubule-associated protein 1 light chain 3(LC3)-phosphatidylethanolamine (PE) conjugation, and ATG5-ATG12 complex, as well as a complex of class III phosphatidylinositol 3-kinases (PI3K) composed of vacuolar protein sorting 34 (VPS34), VPS15, and Beclin 1 (Atg6 in yeast), which can be blocked by the PtdIns3K inhibitors 3-methyladenine (3-MA). Macroautophagy is the most common and best characterized form of autophagy and will be referred to as autophagy thereafter and mainly discussed in the present review

### 2.2. Microautophagy

Microautophagy is a nonselective lysosomal degradative process in mammalian cells, which has been mainly investigated in yeast but is not well defined in mammalian cells [22]. Microautophagy can be induced by nitrogen starvation or rapamycin and plays a crucial role in the maintenance of organellar size, membrane homeostasis, and cell survival under nitrogen restriction. It has been shown that microautophagy is involved in direct engulfment of cytoplasmic proteins or organelles such as long-lived proteins and membrane proteins into the lysosome via invagination [22, 23], which was regulated by a dynamin-related GTPase Vps1p [24].

### 2.3. Chaperone-Mediated Autophagy

Chaperone-mediated autophagy (CMA) is an extremely selective type of autophagy, recognizing the substrate proteins containing specific pentapeptide motif KFERQ via a chaperon protein, heat shock protein 70 (HSP70). Upon recognition, the substrate/chaperone complex is translocated across the lysosome membrane, engulfed, and degraded by lysosomes dependent on the lysosomal membrane protein 2A (LAMP2A). Recently, accumulating studies showed important roles of CMA in Parkinson disease, immunosuppression, and the development of hepatocellular carcinoma [25–27].

## 3. Process of Autophagy

### 3.1. Initiation and Nucleation of Autophagy

Under various conditions of stress like starvation, hypoxia, and oxidative stress, autophagy is initiated by activation of the ULK1 complex (consisting of ULK1, autophagy-related protein 13 (ATG13), RB1-inducible coiled-coil protein 1 (FIP200), and ATG101), which is controlled via phosphorylation by the energy sensor 5-AMP-activated protein kinase (AMPK) and the metabolic integrator mechanistic target of rapamycin complex 1 (mTORC1) [28]. The nucleation is triggered via the activation of class III PI3K complex, which consists of VPS 34 (also known as PIK3 C3), Beclin 1, autophagy-related protein 14-like protein (ATG14L), activating molecule in Beclin 1-regulated autophagy protein 1 (AMBRA1), and general vesicular transport factor (p115), which in turn increases the local concentration of phosphatidylinositide-3-phosphate (PI3P) [29, 30].

### 3.2. Elongation

PI3P produced by the PI3K complex recruits the PI3P effector proteins WD repeat domain phosphoinositide-interacting protein (WIPI2) and zinc-finger FYVE domain-containing protein 1 (DFCP1) to the omegasome. Next, two ubiquitin-like systems activated by WIPI2 and DFCP1 elongate the autophagosome. The first is the ATG12 system, in which ATG5 is covalently conjugated to ATG12 through the actions of atG7 and atG10 and then associates with ATG16L1 to form the ATG12–ATG5–ATG16L complex [31, 32]. The second system results in the binding of PE and ATG8, including microtubule-associated protein 1 light chain 3 (LC3) and *γ*-aminobutyric acid receptor-associated proteins (GABARAPs), which promotes the interaction between the autophagosome and lysosome [33, 34]. In this conjugation reaction, LC3 is converted into LC3-II, a preferred marker of autophagic activity [35, 36]. It is also essential for the closure of the autophagosome [37].

### 3.3. Fusion and Degradation

To be degraded, the autophagosome next fuses with the lysosome and matures into an autolysosome [38], which is regulated by soluble N-ethylmaleimide-sensitive factor attachment protein receptor (SNARE) proteins and homotypic vacuole fusion and protein sorting (HOPS) [39–41]. Once autophagosome–lysosome fusion occurs, the autophagic cargo and the internal autophagosome membrane are digested by lysosomal acidic hydrolases. Finally, the salvaged nutrients are released into the cytoplasm to be recycled.

## 4. Autophagy in Ischemic Stroke

### 4.1. Autophagy in Neurons after Ischemic Stroke

**Figure 1:**
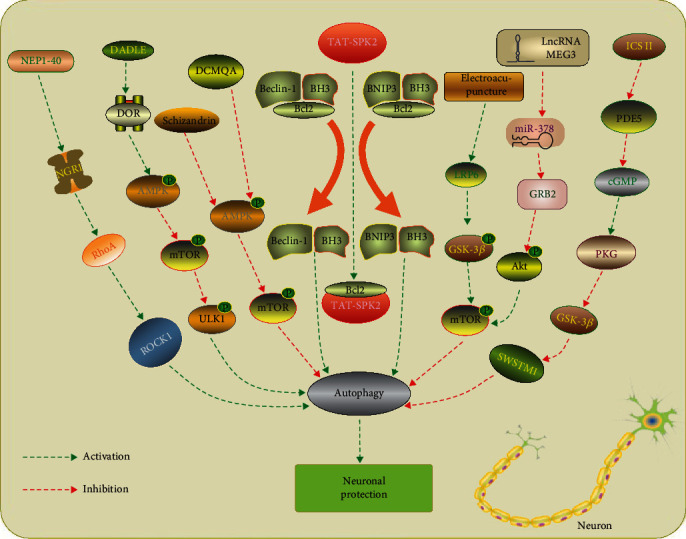
Manipulation of autophagy in neurons after ischemic stroke. NEP1-40 treatment inhibits autophagic activation via NGR1/RhoA/ROCK signaling pathway and attenuates secondary neuronal damage [57]. DADLE has been shown to protect ischemic CA1 neurons by activating delta opioid receptor (DOR)-AMPK-autophagy axis [55]. 4 Schizandrin and DCMQA inhibit neuronal apoptosis via suppression of AMPK/mTOR-mediated autophagy [50, 58]. TAT-SPK2 interacts with Bcl-2 via its BH3 domain, thereby dissociating it from Beclin 1, activating autophagy and protecting neurons against ischemic injury [69]. Electroacupuncture pretreatment induces tolerance to cerebral ischemia by inhibiting autophagy through the inhibition of GSK3*β* [59]. LncRNA MEG3/miR-378/GRB2 axis is involved in neurological functional impairment targeting autophagy in ischemic stroke [60]. Icariside II has been reported to attenuate neuronal injury via inhibiting PKG/GSK-3*β*/autophagy axis [51].

Although accumulating studies indicated that autophagy played a critical role in starvation-induced protein turnover and damaged organelles elimination, the exact role of autophagy in neurons is complex. Under normal conditions, autophagy is maintained at relatively low levels in neurons. Neuron is the most sensitive cells upon ischemia, and several studies have reported the enhanced neuronal autophagy in response to cerebral ischemic injury [42–44] (Figure 1). Using green fluorescent protein (GFP)-fused LC3 transgenic mice, Xie et al. demonstrated that GFP-LC3 fluorescent signals were detected above the ischemic hemisphere (especially in the peri-ischemic area) at 1, 3, and 6 d after transient middle cerebral artery occlusion (tMCAO), with a peak at 1 d. A recent study observed that selective neuronal deletion of the Atg7 (autophagy-related 7) gene prevented hypoxia-ischemia (HI)-induced autophagy, decreased 42% of tissue loss compared to wild-type mice, and reduced neuronal cell death [45]. Astrocyte-derived proinflammatory cytokine interleukin (IL)-17A has been shown to aggravate neuronal ischemic injuries and enhance autophagy oxygen glucose deprivation/reperfusion (OGD/R)-treated neurons. In addition, intracerebroventricular injection of IL-17A neutralizing mAb improved functional recovery in ischemic mice via inhibition of neuronal autophagy through Src-PP2B-mTOR pathway [46]. Zeng et al. demonstrated that knockdown of regulated in development and DNA damage responses 1 (REDD1), a conserved stress response gene [47], reversed the excessive activation of autophagy in neurons, reduced neuronal oxidative stress, and attenuated neuronal cell death upon OGD/R. Moreover, the protective effect of REDD1 suppression could be abolished by blocking the mTOR pathway via rapamycin treatment [48]. The following studies also confirmed the detrimental effects of neuronal autophagy in ischemic stroke [49–55].

Indeed, autophagy has been identified as a double-edged sword in neurons after ischemic stroke [56]. There were also a large number of studies that observed opposite effect of neuronal autophagy in ischemic stroke [57–60]. Our previous study showed that receptor for activated protein kinase C 1 (RACK1), an integral component of ribosomes, was decreased in neurons within penumbra tissue after MCAO. Phosphorylation of RACK1 at T50 reduced infarct size, neuronal death, neuronal tissue loss, and neurobehavioral dysfunction, through promoting Beclin 1 binding in axons and autophagy induction after ischemia [61]. In line with our results, previous studies demonstrated that inhibition of autophagy by 3-MA or Atg7 knockdown enhanced the I-R-induced release of cytochrome c and aggravated ischemia-induced neuronal cell death [9, 62]. In addition, treatment with rapamycin, an mTOR inhibitor, increased autophagy and reduced neuronal death and brain injury in neonatal HI. [63, 64] These results have been confirmed in the following studies in MCAO models [65–68]. A recent study indicated that sphingosine kinase 2 (SPK2) overexpression protected cortical neurons against OGD injury via enhancement of autophagy flux. Moreover, the mechanism underlying autophagy activation induced by SPK2 was that SPK2 interacts with Bcl-2 via its BH3 domain, thereby dissociating it from Beclin 1 complex and activating autophagy. [69] It was also reported that expression of the endogenous NPC1L1 (Niemann–Pick disease type C1-like 1) increased mainly in neurons after MCAO. Treated with NPC1L1 inhibitor ezetimibe (Eze) attenuated neuronal apoptosis and improved long-term neurological recovery in the rat model of MCAO, specifically via activation of the AMPK/ULK1/autophagy pathway [70]. The neuroprotective roles of neuronal autophagy in ischemic stroke have also been confirmed in several studies [71–74].

### 4.2. Autophagy in Microglia after Ischemic Stroke

**Figure 2:**
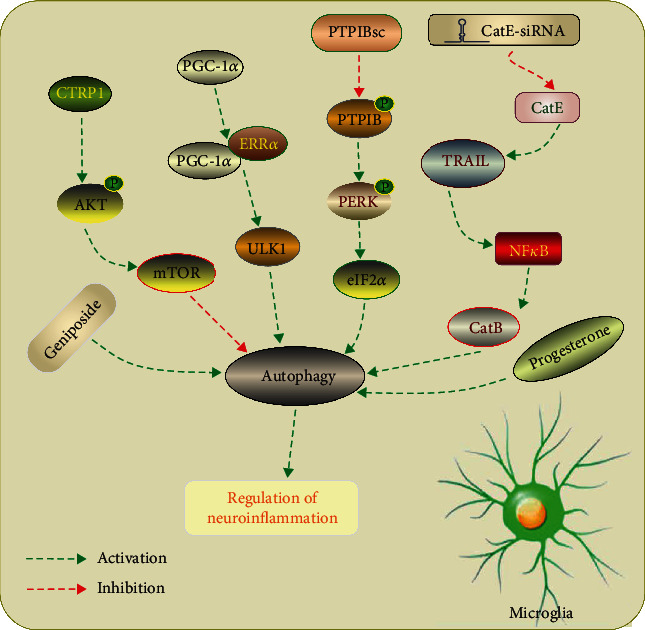
Manipulation of autophagy in microglia after ischemic stroke. CTRP1 inhibits microglia autophagy and inflammation response by regulating the Akt/mTOR pathway after IS [88]. Microglia-specific PGC-1*α* activates autophagy through promoting the expression of ULK1 in an ERR*α*-dependent manner, thereby suppressing neuroinflammation [90]. Inhibition of PTPIB mitigates microglial activation by inhibiting PERK/eIF2*α*-dependent autophagy after ischemic stroke [87]. A proteolytic relay through the early CatE/TRAIL-dependent proteasomal and late CatB-dependent autophagic pathways for NF-*κ*B activation plays an essential role in the neurotoxic polarization of microglia following ischemic stroke [83]. Geniposide and progesterone inhibits NLRP3 inflammasome activation via suppression autophagy in microglial cells in OGD and ischemic brain injury models [92, 93].

Microglia are the major immune cells in brain involved in the postischemic inflammation [75]. Following ischemia, microglial cells were activated with different phenotypes with distinct functions, mainly including M1 and M2 phenotypes [76–80]. The M1 microglia secretes proinflammatory factors, such as interleukin (IL)-6, IL-18, and tumor necrosis factor-alpha (TNF)-*α*, further contributing to severe ischemic injury [81]. Conversely, M2 microglia releases anti-inflammatory mediators, which are responsible for scavenging of debris and secreting trophic factors for brain repair [82]. Recently, there has been increasing evidence that autophagy, especially microglia autophagy, played an important role in the neuroinflammatory response after ischemic stroke (Figure 2).

HI was reported to induce M1 polarization of microglia/macrophages, which contributed to the neuronal death in neonatal mice. The authors also found that excessive CatB-mediated autophagy in microglia cells played an essential role in the neurotoxic polarization of microglia/macrophages following HI injury [83]. Using a permanent middle cerebral artery occlusion (pMCAO) model in mice, Yang et al. observed that autophagy was induced in microglia cells, accompanied with increased inflammatory response. Treatment with 3-MA decreased microglia autophagy and inflammatory response, subsequently decreased infarct size and edema formation, and improved functional recovery [84]. A recent study indicated that baicalein, a biologically active ingredient extracted from the root of Scutellaria baicalensis Georgi, reduced neurobehavioral deficits and decreased brain infarct volume via inhibiting M1 transformation of microglia/macrophage and neuroinflammation. They observed that NF-*κ*B and MAPK signaling pathways were involved in the regulation of microglia/macrophages M1/M2 transformation. Moreover, baicalein also inhibited autophagy via the PI3K/Akt/mTOR signaling pathway [85]. Interestingly, He et al. demonstrated that ischemic stroke activated autophagy signaling in penumbra and reduced CX3CL1 expression on autophagic neurons, finally promoting microglial inflammation. Autophagy inhibitor 3-MA was shown to reverse the microglial inflammatory injury induced by ischemic stroke, whereas treatment with the autophagy inducer Tat-Beclin 1 aggravated the microglial inflammation and cerebral edema [86]. Several recent researches also inhibition of microglia autophagy might be a potential therapeutic strategy for ischemic stroke treatment [87–89].

Although accumulating studies showed a detrimental role of microglia autophagy in ischemic stroke, Han et al. showed that overexpression of microglia-specific PGC-1*α* significantly decreased neurologic deficits after ischemic injury, with reduced NLRP3 activation and proinflammatory cytokine production via activation autophagy [90]. Moreover, a recent study demonstrated that activation of autophagy in microglial cells was time dependent and inhibition of microglia autophagy promoted neuroinflammation. They observed that the autophagic flux was induced at early phase of OGD/R (12h, 24h, and 48h after OGD/R) whereas inhibited at the late phase of OGD/R (72h after OGD/R). Xia et al. also demonstrated that inhibition of autophagic flux induced M1 microglial phenotype of microglial cells with increased tumor necrosis factor-alpha (TNF-*α*), inducible nitric oxide synthase (iNOS), and cyclooxygenase-2 (COX-2). In addition, autophagy participated in the alternation of microglial phenotype via modulating NF-*κ*B pathway and activity of CREB [91]. The neuroprotective roles of microglia autophagy in ischemic stroke have also been indicated in other studies [92, 93].

### 4.3. Autophagy in Astrocytes after Ischemic Stroke

Astrocytes are the most abundant cell type within the central nervous system, and they play essential roles in maintaining normal brain function, including structural support, neuronal metabolism, maintenance of the extracellular environment, and neurotransmitter synthesis [94–96]. Following ischemic stroke, astrocytes perform multiple functions both detrimental and beneficial. Astrocytic inflammatory response aggravates the ischemic lesion, and the glial scar obstructs axonal regeneration during the late phase after stroke [97–99]. However, astrocytes also provide benefit for angiogenesis, synaptogenesis, and axonal remodeling [100–102].

Autophagy in astrocytes was induced by cerebral ischemia as evidenced by the increased expression of autophagy and autolysosome-related proteins, including microtubule-associated protein 1 light chain 3 (LC3-II), Beclin 1, lysosome-associated membrane protein 2 (LAMP2), and lysosomal cathepsin B. Furthermore, inhibition of autophagy by 3-MA attenuated OGD-induced death of astrocytes and increased GFAP-positive cells in the ischemic cortex 12 h following pMCAO. [103] Similarly, Zhou et al. showed that autophagy was activated in ischemic astrocytes and pharmacological or genetic inhibition of autophagy reversed OGD-induced release of cathepsin B and L from the lysosome to the cytoplasm and activation of caspase-3 in the astrocytes, decreased OGD-induced increase in lysosomal membrane permeability, and subsequently reduced OGD-induced astrocytic cell death and infarction volume in rats [104]. Receptor-interacting protein 1 kinase (RIP1K) is a crucial mediator of necroptosis after ischemic stroke. Ni et al. demonstrated that OGD or pMCAO condition increased the expression of RIP1K (receptor-interacting protein 1 kinase), RIP3K, and RIP1K–RIP3K complex. They further found that pharmacological or genetic inhibition of RIP3K attenuated astrocytic necrotic cell death in the ischemic cortex, reduced infarct volume, and improved neurological deficits in the MCAO model. In addition, a combination of Nec-1 (pharmacological inhibitor of RIP3K) and an inhibitor of autophagy produced an enhancement protective effect on astrocytic cell death after ischemic stroke, suggesting that activation of the autophagic-lysosomal pathway was involved in the RIP1K-mediated ischemic astrocytic necroptosis [105]. Using a circRNA microarray, Han et al. found that circular RNA Hectd1 (circHectd1) levels were significantly increased in both ischemic brain tissues in transient MCAO mouse stroke models and in plasma samples from acute ischemic stroke patients. CircHectd1 acts as an endogenous RNA sponge and binds miR-142 to promote astrocytic autophagy via the downstream target TIPARP (TCDD inducible poly [ADP-ribose] polymerase), resulting in astrocyte activation in stroke. Knockdown of circHectd1 expression significantly attenuated astrocytic autophagy, decreased infarct areas, attenuated neuronal deficits, and ameliorated astrocyte activation in tMCAO mice [106]. A recent study also confirmed the detrimental effects of astrocyte autophagy in ischemic stroke [107].

**Figure 3:**
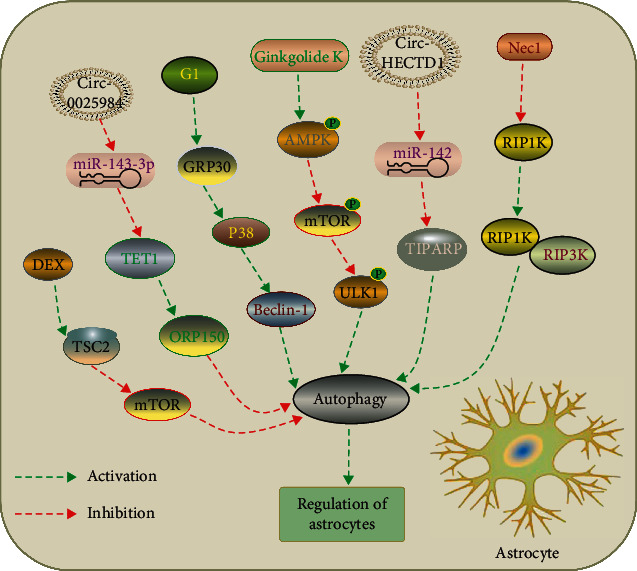
Manipulation of autophagy in astrocytes after ischemic stroke. DEX exerts a neuroprotection against OGD-induced astrocytes injury via activation of astrocytes autophagy by regulating the TSC2/mTOR signaling pathway [108]. Circular RNA 0025984 protects astrocytes from ischemic injury via inhibition of autophagy by targeting the miR-143-3p/TET1/ORP150 pathway [107]. G1 treatment restores autophagy in astrocytes via activation of G protein-coupled receptor 30 (GRP30) and protected neurons after ischemic stroke [109]. Ginkgolide K promotes astrocyte proliferation and migration after oxygen-glucose deprivation via inducing protective autophagy through the AMPK/mTOR/ULK1 signaling pathway [110]. Knockdown of Circular RNA HECTD1 inhibits astrocyte autophagy via MIR142/TIPARP axis, resulting in inhibition of astrocyte activation after cerebral ischemic stroke [106]. Nec-1, a specific inhibitor of RIP1K, decreased RIP1K–RIP3K complex formation and inhibited autophagy, thereby attenuating astrocytic necrotic cell death in the ischemic cortex [105].

However, several studies observed the contrary results [108–110] (Figure 3). Back et al. demonstrated that activation astrocytic GPR30 (G protein-coupled receptor 30) restored autophagy in glutamate-induced astrocyte via p38 mitogen-activated protein kinase (MAPK) signaling pathway, inhibited reactive astrogliosis, and decreased inflammatory cytokine release, finally reducing the neurological deficit scores and the infarct volume after MCAO [111]. Interestingly, a recent study demonstrated that upregulation of autophagy flux of astrocytes decreased neuronal apoptosis after exposed to OGD/R. Moreover, induction of astrocyte autophagy by AAV-GFAP-ATG7 in vivo improved neurological recovery in MCAO model in mice, suggesting that astrocytic autophagy might contribute to endogenous neuroprotective and neurological recovery after stroke [67].

### 4.4. Autophagy in Oligodendrocytes after Ischemic Stroke

Oligodendrocytes are abundant in both gray and white matter of the brain and are the only cells able to form the myelin sheath in the CNS [111, 112]. However, most of oligodendrocytes are dead after ischemic stroke, which are associated with neurological defect [113, 114]. In response to ischemic injury, oligodendrocyte progenitor cells, derived from NPCs (neural progenitor cells) in the SVZ (subventricular zone), proliferate and differentiate into oligodendrocytes to form new myelin sheaths [115, 116]. A recent study demonstrated that autophagy played a critical role in the oligodendrocyte differentiation, survival, and proper myelination. Mice with conditional OPC/OL-specific deletion of Atg5 beginning on postnatal day 5 developed a rapid tremor and died around postnatal day 12, with apoptotic death of OPCs and reduction in differentiation and myelinated axons. In addition, induction of autophagy in OPCs promoted myelination of DRG neurons in cocultures, which was blocked with autophagy inhibition [117]. Current evidence indicated that oligodendrocyte autophagy played an important role in neurodegenerative disease and spinal cord injury [118–121]. However, studies involved in autophagy of oligodendrocytes after ischemic stroke were limited.

### 4.5. Autophagy in Brain Microvascular Endothelial Cells and BBB Permeability after Ischemic Stroke

**Figure 4:**
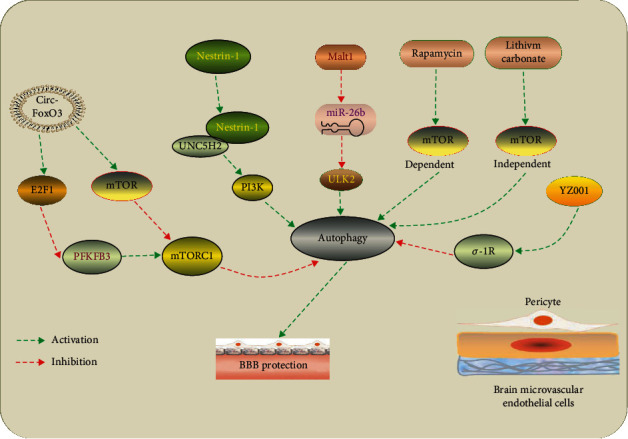
Manipulation of autophagy in brain microvascular endothelial cells after ischemic stroke. Circ-FoxO3 inhibits mTORC1 activity mainly by sequestering mTOR and E2F1, thus promoting autophagy to clear cytotoxic aggregates for improving BBB integrity [130]. Netrin-1 ameliorates BBB impairment secondary to ischemic stroke by activating PI3K-mediated autophagy depending on UNC5H2 receptor [129]. LncRNA Malat1 protects brain microvascular endothelial cells (BMECs) against ischemic injury by sponging miR-26b and upregulating ULK2 expression, thereby promoting BMEC autophagy [131]. Rapamycin and lithium carbonate pretreatments improve BBB integrity after ischemic stroke through induction of mTOR-dependent and mTOR-independent autophagy, respectively [127]. YZ001, a new *σ*-1R agonist, enhances pericyte survival via inhibition of autophagy in ischemic stroke [136].

Blood-brain barrier (BBB), composed of brain microvascular endothelial cells (BMVECs), perivascular astrocytes, neurons, and pericytes, regulates substance influx and efflux to maintain a normal physiology of brain. Among them, BMVECs, connected by tight junctions, play a critical role in the maintaining BBB integrity [122, 123]. It has been reported that the impairment of BBB integrity is a common pathological feature after ischemic stroke, which subsequently aggravates brain edema and neuronal injury [124, 125]. Accumulating studies focused on the role of autophagy in BBB dysfunction in ischemic stroke (Figure 4). FAK-phosphorylation-dependent activation of autophagy served as an endogenic protective response to endothelial injury in vitro induced by methylglyoxal (MGO), a reactive dicarbonyl which could exacerbate ischemia-induced BMEC injury. The beneficial effect of autophagy was also observed in the MCAO model [126]. We recently observed that enhancement of autophagy by rapamycin attenuated BMVEC apoptosis after OGD/R, whereas which was increased via autophagy inhibition by 3-methyladenine (3-MA). Moreover, autophagy induced by rapamycin and lithium carbonate also attenuated the decrease expression of ZO-1, a kind of tight junction protein, and promoted the distribution of ZO-1 on cell membranes, indicating a beneficial effect of BMVEC autophagy on BBB integrity [127]. This was also supported by Urbanek et al., who showed that treatment with rapamycin increased the viability of human umbilical vein endothelial cells (HUVECs) after OGD, whereas knockdown of Beclin 1 by siRNAs attenuated the autophagy and reduced cell viability of HUVECs [128]. In addition, Yu et al. showed that Netrin-1, an axonal guidance molecule, enhanced autophagy activity via PI3K pathway depending on UNC5H2 receptor and attenuated BBB impairment induced by ischemic stroke by promoting tight junction function and endothelial survival [129]. Moreover, recent studies also suggested that endothelial autophagy might contribute to blood-brain barrier protection after ischemic stroke [130, 131].

On the other hand, increasing evidence suggested a detrimental role of endothelial autophagy in BBB dysfunction in ischemia. After focal ischemia in the barrel cortex, increased autophagy-like cell death (including in BMVECs and neurons) and BBB impairment were observed in NF-*κ*B p50 knockout mice, which could be reversed by autophagic inhibition [132]. Zhang et al. demonstrated that enhanced autophagy in rats with hyperglycemia contributed to the autophagy-lysosome-mediated degradation of ZO-1 and was, at least partially, responsible for the BBB dysfunction after MCAO [133]. Similar results were observed in a recent study [134]; using in vitro OGD model in mouse BMVECs, Liu et al. showed that inhibition of autophagy or the fusion of autophagosome with lysosome blocked OGD-induced claudin-5 degradation, subsequently attenuated endothelial barrier disruption [135]. Taken together, these results showed a detrimental role of endothelial autophagy in BBB dysfunction after ischemic stroke [136].

## 5. Autophagy: A Double-Edged Sword in Ischemic Stroke

Emerging evidence suggests that autophagy serves as a double-edged sword in several human diseases, such as neurodegenerative diseases [137], cancer [138], and infectious diseases [139]. Likewise, as all the evidence above shows, either the absence of autophagy or excessive autophagy may be detrimental to the outcomes of cerebral ischemia. However, currently there is no unified theory of whether autophagy plays a beneficial or detrimental role in ischemic stroke. Based on studies over past few years, there were two potential factors that determine the role of autophagy in ischemic stroke, the levels of autophagy, and the time of autophagy.

Kang et al. demonstrates that levels of autophagy are critical for the fate of cells, moderate or adaptive autophagy can promote survival, whereas excessive (maladaptive) levels of autophagy promote cell death [140]. This speculation may be supported by an in vitro OGD model, the results of which show the dual role of 3-MA at different time points of reoxygenation [141]. Autophagy inhibition by 3-MA at 24 hours prior to reperfusion triggers a higher neuronal death rates, whereas at 48 and 72 hours after reperfusion, 3-MA treatment significantly protected neurons from death. It is possible that transient OGD induces adaptive autophagy that eliminates damaged organelles to rescue neurons, while prolonged OGD triggers excessive autophagy leading to neurons death and switching its role from protection to deterioration.

In addition, the time at which autophagy induced is another key variable in the balance between protective or detrimental autophagy. It has been shown that in the treatment with rapamycin or 3-MA 20 minutes before hypoxia-ischemia, rapamycin reduces necrotic cell death and decreases brain injury via autophagy induction, while 3-MA inhibits autophagy and promotes neuronal death [63, 64]. Conversely, Puyal et al. indicated that postischemic intracerebroventricular injections of 3-MA significantly reduced the lesion volume (by 46%) [44]. This was supported by a study in which administration of 3-MA suppressed autophagy induced by preconditioning and attenuated the neuroprotection of preconditioning against cerebral ischemia. In contrast, pretreatment with rapamycin mimicked the neuroprotective effect of ischemic preconditioning [142]. Gao et al. reported that induction of autophagy by rapamycin attenuate the neuroprotective effects of ischemic postconditioning in the MCAO model [143], whereas blocking the AKT-GSK3*β* signaling pathway prior to ischemic postconditioning suppressed the autophagy and neuroprotection of ischemic postconditioning [144]. Qi et al. also found that the involvement of Akt mediated Bcl-2 phosphorylation and thereby Bcl-2/Beclin1 complex disruption played a crucial role in triggering autophagy and were essential for the neuroprotective effects of ischemic postconditioning [145]. Based on these findings, manipulation of autophagy at different time point after ischemic stroke may also determine its role, suggesting that adaptive autophagy induced at early stage may provide a prosurvival effect but prolonged and excessive autophagy causes nerve injury at late stage. Moreover, manipulation of autophagy in specific type of cells by cell-specific deletion of autophagy-related genes might be a reason for discovering contradictory results, because interfering with autophagy in single cell type might not prove it decisively contributes to beneficial or harmful results after ischemic stroke [45, 146]. Finally, the controversial effects of autophagy may also be due to the different model types, different types of selective autophagy, different means of intervention, or even sex-specific differences in experimental animal. [147–149]

## 6. Potential Therapeutic Strategies for Ischemic Stroke Targeting Autophagy Manipulation

According to the aforementioned studies, therapeutic strategies targeting autophagy modulation may be a possible approach in the management of IS. To date, multiple potential therapeutic agents have been explored [150–154]. These agents modulate different processes of autophagy including (A) inducing adaptive autophagy and (B) inhibiting excessive autophagy following IS. A variety of compounds have been shown to induce adaptive autophagy. Among these compounds, rapamycin is widely examined in the management of IS via inhibition of mTOR. Recent investigations indicated that administration of rapamycin in rodents undergoing MCAO could diminish infarct volume, reduce neuronal injury, and improve neurological recovery [68, 155, 156]. Rapamycin also has been reported to reduce endothelial cell death and improve BBB permeability in an MCAO model [127, 157]. A recent systematic review involving 17 publications demonstrated that rapamycin significantly reduced infarct volume by 21.6% and improved in neurobehavioral scores by 30.5%. Interestingly, lower doses of rapamycin showed greater efficacy at reducing infarct volume than higher doses, which is potentially due to an optimal level of autophagy activation with low dose of rapamycin [156, 158, 159]. Resveratrol, a common dietary polyphenol, has been shown to extend the clinical therapeutic window of r-tPA for stroke patients [160]. He et al. reveled that resveratrol alleviated cerebral I/R injury and reduced infarct size [161], which was consistent with another study [162]. For inhibition of excessive autophagy, several studies demonstrated that dexmedetomidine (DEX) was capable of rendering neuroprotection after ischemic stroke via inhibition of excessive autophagy. In the primary cultured neurons in oxygen-glucose deprivation (OGD) model and tMCAO model, DEX protected mouse brain from ischemia-reperfusion injury via inhibition of neuronal autophagy by upregulation of HIF-1*α* [52]. Moreover, DEX has been reported to reduce autophagy effect and improve learning and memory dysfunction in rodent MCAO model [163]. A recent study showed that regulation of miR-199 was a potential mechanism by which DEX inhibited autophagy and promoted neurological recovery after ischemic stroke [164]. Propofol administration reduced the infarct size and improves the outcome of acute ischemic stroke [165, 166]. Recent studies demonstrated that propofol protects against cerebral ischemia/reperfusion injury via inhibition of excessive autophagy through regulation of mTOR/S6K1 or long noncoding RNA SNHG14 [167, 168]. Melatonin, an endogenous hormone, was demonstrated to significantly alleviate cerebral infarction, brain edema, and neuronal apoptosis after ischemic stroke inhibited ER stress-induced excessive autophagy [169]. Recently, Gao et al. showed Icariside II, one of the main active ingredients of Epimedii (a traditional Chinese medicine), protecting neurons in OGD and rodent MCAO models via inhibiting excessive autophagy through interfering with the PKG/GSK-3*β* signaling pathway [51].

Although accumulating research has been shown that agents targeting the autophagy pathway have therapeutic potential for IS, there are several limitations need to be considered. Firstly, the possible side effects of the agents should be considered. Besides enhancing autophagy, mTORC1 inhibition blocks protein and nucleotide synthesis, inhibits cell proliferation, and inhibits metabolism [170]. Long-term rapamycin administration may cause immunosuppression, and glucose intolerance due to mTORC1 is acutely sensitive to rapamycin whereas mTORC2 chronically sensitive to rapamycin in vivo [171]. In addition, recent studies mainly focused on the effect of autophagy regulation on neuronal cell damage but less on cell growth and secretion in ischemic stroke. Finally, the outcome of treatment targeting autophagy is not only associated to the degree of autophagy, but also to the administration time of autophagy regulators and drug dose as well as the administration route.

## 7. Conclusions and Future Perspectives

As aforementioned above, accumulating evidence demonstrates that autophagy plays a critical role in the pathological process of ischemic stroke, which may provide a potential therapeutic target for ischemic stroke. However, there are many unanswered questions that should be carefully and critically addressed by future studies to translate autophagy-based stroke therapies to the clinic. Whether there are noncanonical pathways that initiate maladaptive autophagy which is detrimental to neuronal cell survival? Autophagy after ischemic stroke involves a variety of pathways, but by which mechanisms regulation the degree of autophagy? Excessive autophagy is characterized by accumulated autophagosomes, while the underlying molecular mechanisms are still unclear. How to manipulate cell-specific autophagy in a selective manner without activating unwanted cell death signaling pathways? Given that IS-induced autophagy has both beneficial and detrimental effects, exploring the most likely influencing factors such as optimal time point for autophagy manipulation should be considered. Finally, the last and most important point is that translation of this therapeutic strategy from the laboratory to the clinic should be accompanied by robust preclinical studies in appropriate cell culture and animal models.

## Data Availability

The data and materials can be obtained by contacting the corresponding author.
